# Expression of matrix metalloproteinases-8 and -9 and their tissue inhibitor in the condyles of diabetic rats with mandibular advancement

**DOI:** 10.3892/etm.2014.1984

**Published:** 2014-09-22

**Authors:** XIAOHUAN ZHONG, HUIXIN WANG, XINCHUN JIAN

**Affiliations:** Department of Stomatology, Xiangya Hospital, Central South University, Changsha, Hunan 410008, P.R. China

**Keywords:** type 1 diabetes mellitus, condyle, matrix metalloproteinase-8, matrix metalloproteinase-9, tissue inhibitor of metalloproteinase-1

## Abstract

The present study aimed to evaluate the effects of type 1 diabetes mellitus on the condylar response during treatment with a functional appliance. Sprague-Dawley rats were divided into 3 groups, normal (NG), diabetes (DG) and diabetes with insulin-treatment (TG). Bite-jumping appliances were fitted to the rats in the experimental groups. At 7, 14, 21 and 28 days following fitting, animals were sacrificed and condyles were excised and processed using routine histological techniques. The expression of matrix metalloproteinase (MMP)-8, MMP-9 and tissue inhibitor of metalloproteinase-1 (TIMP-1) was detected using immunohistochemical analysis. Mandibular advancement increased the expression levels of MMP-8 (peaked on day 28), MMP-9 (peaked on day 21), TIMP-1 (peaked on days 21 and 28) and the ratio of MMP-8 to TIMP-1 and MMP-9 to TIMP-1. In the DG, diabetes decreased the expression levels of MMP-8 and MMP-9 induced by mandibular advancement and increased the expression levels of TIMP-1 compared with that of the NG. The ratio of MMP-8 to TIMP-1 and MMP-9 to TIMP-1 also showed a significant decrease in the DG compared with that of the NG. A recovery of these parameters was observed in the TG. Diabetes significantly altered the condylar response, which was triggered by mandibular advancement, and weakened subsequent bone deposition. The results from the TG were not significantly different from that of the NG.

## Introduction

Class II malocclusion is one of the most common orthodontic problems. Mandibular retrognathia is the major etiology of class II malocclusion (1). A variety of functional appliances have been used to stimulate mandibular growth in adolescence with mandibular retrognathia. Mandibular advancement achieved by the fitting of a functional appliance may promote condylar remodeling and subsequently accelerate mandibular growth (2,3).

Type 1 diabetes mellitus, which is characterized by a lack of insulin production, accounts for 5–10% of diabetes and a peak incidence occurs at 10–14 years of age (4,5). Previous studies have shown that a deficiency of insulin may alter the mechanical response of alveolar bone during orthodontic tooth movement in animal models, while insulin treatment normalized this abnormal response (6,7). However, whether the deficiency of insulin in patients with type 1 diabetes mellitus affects condylar remodeling during treatment with functional appliances remains unclear.

A previous study identified that insulin can exert a pronounced effect on the differentiation of cartilage precursor cells into chondroblasts and chondrocytes (8), and promote collagen II and proteoglycan synthesis in chondrocytes (9,10). Researchers also found that the disturbance of glucose metabolism induced by an insulin disorder may negatively affect articular cartilage (11), while insulin treatment ameliorated cartilage degeneration in an animal model of osteoarthritis (12). In addition, recent studies have shown that the insulin receptor can be detected on human cartilage and chondrocytes (13). These results indicate that insulin exerts a direct effect on articular cartilage metabolism.

The aim of the present study was to elucidate the effects of type 1 diabetes on the condylar response during treatment with a functional appliance by analyzing the expression levels of matrix metalloproteinase (MMP)-8, MMP-9 and tissue inhibitor of metalloproteinase-1 (TIMP-1).

## Materials and methods

### Animals and the experimental diabetes and mandibular advancement models

Seventy-five male Sprague-Dawley rats (age, 3 weeks; weight, 60–70 g) were purchased from the Experimental Animal Center of the Third Xiangya Hospital (Changsha, China) and randomly divided into 3 groups: normal (NG), diabetes (DG) and diabetes with insulin-treatment (TG) (n=25 per group). All animals were treated according to the ethical regulations for animal experiments defined by the Ethics Committee of the Central South University (Changsha, China). Type 1 diabetes was induced in rats (DG and TG) by intraperitoneal injection of 85 mg kg^−1^ streptozotocin (STZ; Sigma, St. Louis, MO, USA) freshly dissolved in citrate buffer (0.1 mol/l; pH 4.5), while the NG was injected with an equivalent volume of citrate buffer. The rats were fasted for 12 h prior to STZ injection. Three days following induction, blood samples were collected from the tail vein and the plasma glucose levels were evaluated using a glucose-oxidase enzymatic method (Accu-Chek Performa; Roche Diagnostics GmbH, Mannheim, Germany). STZ-injected rats were considered to have diabetes if glucose levels were >300 mg/dl after 8 h of fasting. The injection of STZ was repeated when glucose levels of <300 mg/dl were detected. Determination of glucose levels was repeated once a week in the non-insulin treated groups. The rats in the TG received daily hypodermic injections of premixed insulin consisting of insulin N and R (Novolin 30R; Novo Nordisk, Bagsvaerd, Denmark) following the successful establishment of the diabetic animal model. Plasma glucose levels were measured twice a day. The dose of insulin was individually adjusted to maintain random blood glucose levels of 90–190 mg/dl.

**Figure 1:**
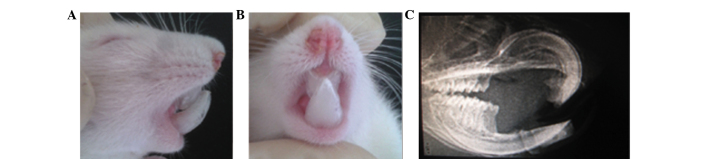
(A) Lateral and (B) frontal views and (C) a radiograph following fitting of a functional applicance showed vertical and anterior displacement of the mandible.

A functional appliance to advance mandible growth was applied to the rats in the experimental groups under anesthetic (10% chloral hydrate, 0.33 ml/100 g of body weight) on the day 1 of week 3 of the experiment. No appliance was fitted to the control animals. The bite-jumping appliance used in this study was constructed according to the method used by Xiong *et al* (14) (Fig. 1). The appliances were worn 24 h per day and resulted in an anterior advancement of 3–4 mm and a vertical displacement of 1–2 mm of the mandible.

Rats were anesthetized and decapitated at 0, 7, 14, 21 and 28 days following mandibular advancement. Condyles were fixed in the 4% paraformaldehyde for 24 h at 4°C and decalcified for 1 week in EDTA at 4°C. The demineralized tissues were dehydrated and embedded in paraffin and the tissue blocks were cut into 5-μm thick sagittal serial sections.

### Histology and histomorphometry

**Figure 2:**
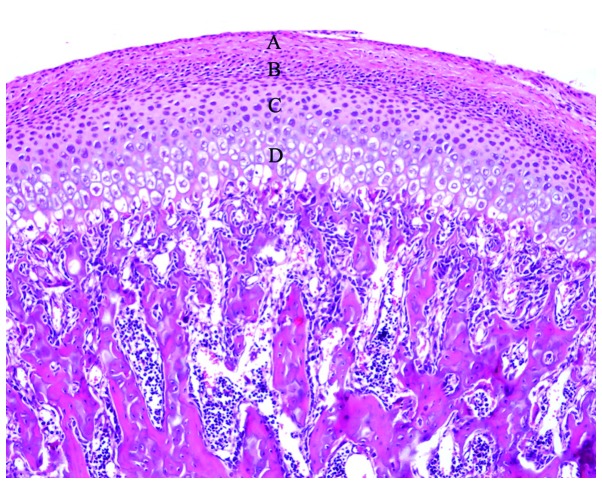
Histological overview of the central section of the mandibular condyle. Hematoxylin and eosin staining with 100-fold enlargement with division of layers. A, articular layer; B, proliferative layer; C, chondrogenic layer; D, hypertrophic layer.

The morphological conditions of the condyles were observed with hematoxylin and eosin staining. The condylar cartilage was divided into four layers for measurement (Fig. 2), which have been previously characterized and described (15). Layer thickness was measured using the method by Ghafari and Degroote (16).

### Immunohistochemical assay

Immunohistochemical reagents were obtained from Wuhan Boster Biological Technology, Ltd. (Wuhan, China). Following deparaffinization, sections were treated with antigen retrieval buffers (compound enzyme digestive juice) for 10 min at room temperature, washed twice in 0.01 M phosphate-buffered saline (PBS) and immersed in 3.0% H_2_O_2_ for 5 min to block endogenous peroxidase activity. Sections were washed twice in 0.01 M PBS, exposed to blocking buffer with 3% goat serum/PBS for 60 min and reacted with rabbit anti-MMP-8, -MMP-9 and -TIMP-1 antibodies overnight at room temperature. The dilution ratio was 1:75, 1:150 and 1:150, respectively. Following rinsing with PBS three times, sections were incubated with goat anti-rabbit secondary antibody conjugated to horseradish peroxidase (dilution, 1:400) for 2 h. Sections were then rinsed with PBS three times and stained with 3,3-diaminobenzidine for 3–5 min at room temperature. PBS was as a negative control.

### Statistical analysis

Results are presented as the mean ± standard deviation. The statistical analysis of the results was performed using one-way analysis of variance followed by Scheffe’s test for multiple comparisons. P<0.05 was considered to indicate a statistically significant difference.

## Results

### Histological observations

**Figure 3:**
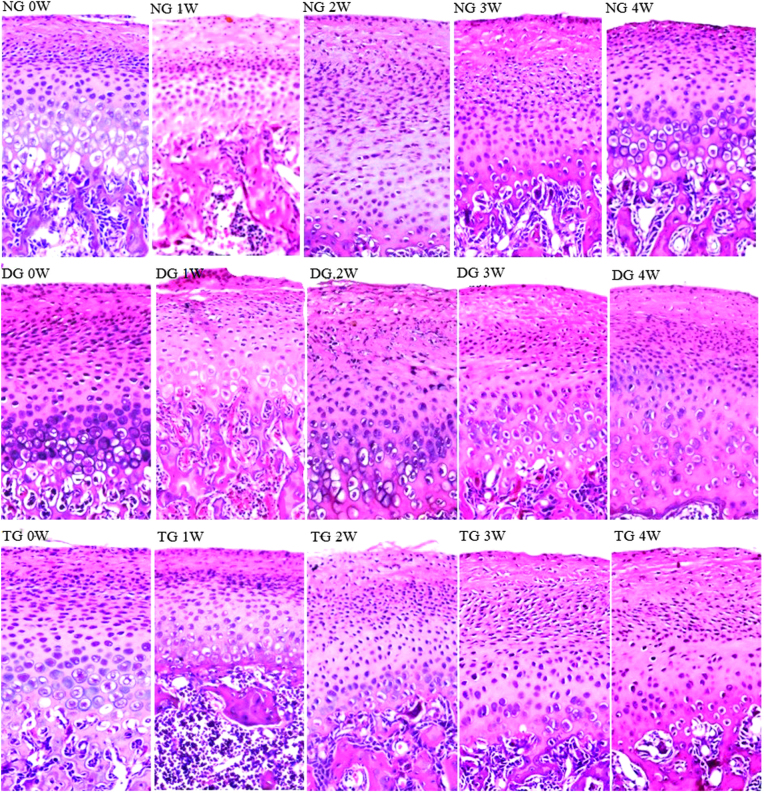
Histological overview of the central section of the mandibular condyle in N, D and T at 0–4 weeks. Hematoxylin and eosin staining with 100-fold enlargement with division of layers. N, normal group; D, diabetes group; T, diabetes with insulin-treatment group.

**Figure 4:**
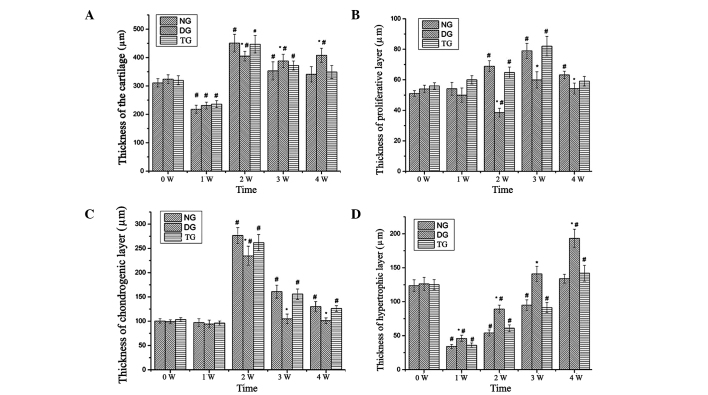
Measurement of the thickness of the (A) condylar cartilage, and (B) proliferative (C), chondrogenic (D) and hypertrophic layers in NG, DG and TG. NG, normal group; DG, diabetes group; TG, diabetes with insulin-treatment group.

Histological analyses of the samples revealed a difference in condylar layer thickness between the DG and NG following mandibular forward positioning (Figs. 3 and 4). Total thickness of the cartilage in the DG was less than that of the NG at day 14 (P<0.05), which was largely indicated by the decreased thickness of the proliferative layer and chondrogenic layer. However, in the DG, the cartilage was thicker than that of the NG at days 21 and 28 (P<0.05), which was largely determined by an increased thickness of the hypertrophic layer, and thinner proliferative and chondrogenic layers than that of the NG. There were no significant differences in histological morphous between the NG and the TG.

### Immunoreactivity of MMP-8 and 9 and TIMP-1

**Figure 5:**
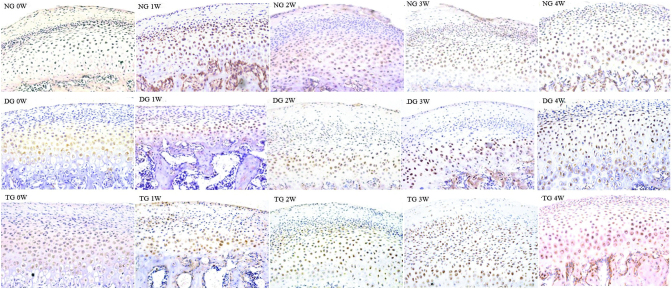

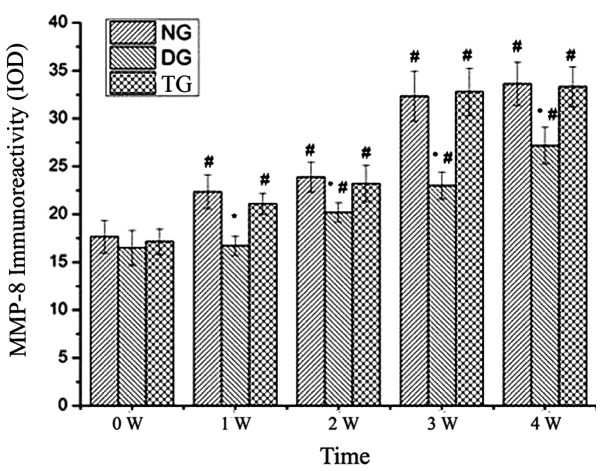
Immunohistochemical staining for MMP-8 in the condylar cartilage in NG, DG and TG (original magnification, ×200). Data are expressed as the mean ± standard error of the mean. ^*^P<0.05, vs. respective experimental group. ^#^P<0.05, vs. DG and TG. NG, normal group; DG, diabetes group; TG, diabetes with insulin-treatment group; MMP-8, matrix metalloproteinase-8.

**Figure 6:**
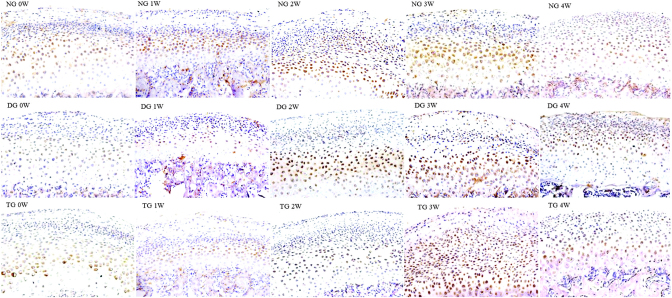

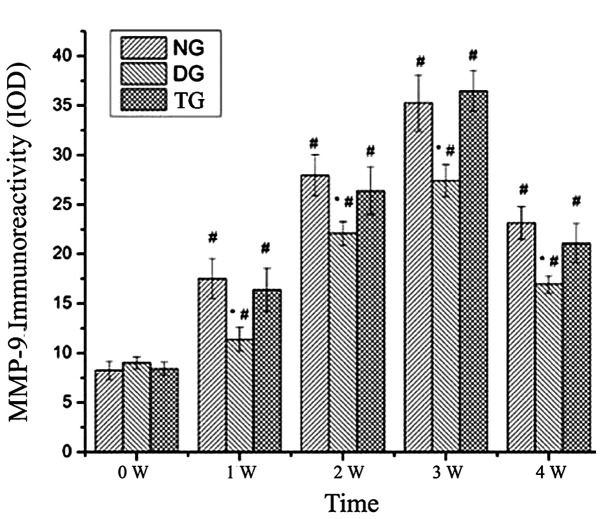
Immunohistochemical staining of MMP-9 in the condylar cartilage in NG, DG and TG (original magnification, ×200). Data are expressed as the mean ± standard error of the mean. ^*^P<0.05, vs. respective experimental group. ^#^P<0.05, vs. DG and TG. NG, normal group; DG, diabetes group; TG, diabetes with insulin-treatment group; MMP-9, matrix metalloproteinase-9.

Positive immunohistochemical staining for MMP-8 was observed in cells in all condylar layers (Fig. 5A). Following mandibular forward positioning, the expression levels of MMP-8 increased during the experiment and reached a peak on day 28 in the DG and NG (P<0.05). However, expression was significantly decreased in the DG compared with that of the NG (P<0.05) (Fig. 5A and B). Positive immunohistochemical staining for MMP-9 was observed mainly in the proliferation and chondrogenic layers in the DG and NG prior to mandibular advancement (Fig. 6A). Following mandibular forward positioning, the expression levels of MMP-9 increased throughout the experimental period and peaked at day 21 in the NG and DG (P<0.05). However, expression was significantly decreased in the DG compared with that of the NG (P<0.05). Positive immunohistochemical staining of MMP-9 was observed predominantly in the proliferation and chondrogenic layers at days 7 and 14, and in the proliferation, chondrogenic and hypertrophic layers at days 21 and 28 in the NG (Fig. 6A and B).

**Figure 7:**
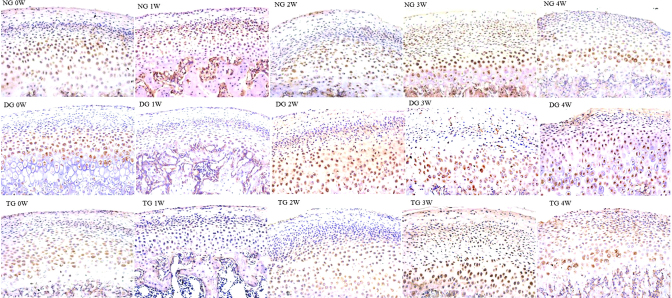

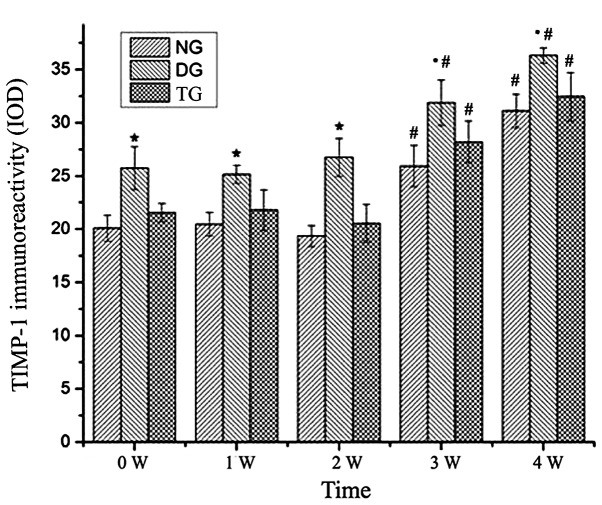
Immunohistochemical staining of TIMP-1 in the condylar cartilage in NG, DG and TG (original magnification, ×200). Data are expressed as the mean ± standard error of the mean. ^*^P<0.05, vs. respective experimental group. ^#^P<0.05, vs. DG and TG. NG, normal group; DG, diabetes group; TG, diabetes with insulin-treatment group; TIMP-1, tissue inhibitor of metalloproteinase-1.

Positive immunohistochemical staining for TIMP-1 was observed mainly in the proliferation, chondrogenic and hypertrophic layers (Fig. 7A). Following mandibular forward positioning, TIMP-1 expression levels were relatively stable in the NG and DG at days 7 and 14, whereas an increase was observed at days 21 and 28 in the two groups (P<0.05). There was a significant difference in the expression levels of TIMP-1 between the NG and DG at days 21 and 28. Diabetes significantly increased the expression levels of TIMP-1 (P<0.05) (Fig. 7A and B).

**Figure 8:**
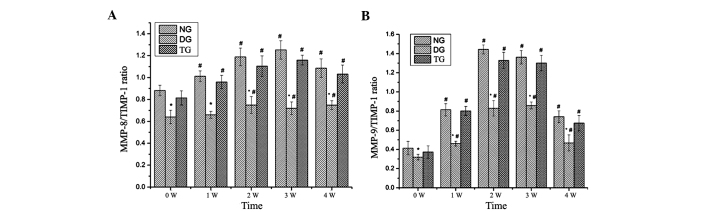
Ratio of (A) MMP-8 to TIMP-1 and (B) MMP-9 to TIMP-1 in the condylar cartilage in NG, DG, and TG. Data are expressed as the mean ± standard error of the mean. ^*^P<0.05, vs. respective experimental group. ^#^P<0.05, vs DG and TG. TIMP-1, tissue inhibitor of metalloproteinase-1; MMP, matrix metalloproteinase; NG, normal group; DG, diabetes group; TG, diabetes with insulin-treatment group.

The ratio of MMP-8 to TIMP-1 increased and peaked at day 21 in the NG, while the ratio of MMP-8 to TIMP-1 increased and peaked on day 14 in the DG. The ratio of MMP-9 to TIMP-1 peaked on day 14 and then gradually declined in the NG, while it reached a peak on day 21 and then declined gradually in the DG. Experimental diabetes significantly decreased the ratio of MMP-8 to TIMP-1 and MMP-9 to TIMP-1 compared with that of the NG (P<0.05) (Fig. 8A and B).

Reversal of the diabetic state using insulin treatment resulted in a recovery of the aforementioned parameters. There were no significant differences in the parameters between the NG and TG (P>0.05) (Figs. 5,6 and 7).

## Discussion

Besides heredity, the growth and development of the condylar cartilage is thought to be induced by external stimuli, including mandibular advancement, hormones and growth factors (17). Insulin can exert a direct effect on chondrocytes and the articular cartilage (8–13), therefore it has been hypothesized that type 1 diabetes mellitus may affect the condylar response during functional appliance treatment. However, the mechanisms involved have yet to be fully elucidated. The results of the present study reveal a significant alteration in the condylar cartilage response of diabetic animals to mandibular advancement.

In the growth and development of the condylar cartilage, hypertrophic condylar cartilage matures to a nonhypertrophic stage. The sequence of this begins with differentiation of mesenchyme cells into chondrocytes in the proliferative layer followed by hypertrophy in the hypertrophic layer and circumferential calcification of the cartilage matrix. The calcified matrix is resorbed and the majority of the matrix in the hypertrophic layer breaks down, which creates space for vascular invasion. The invading capillaries are accompanied by osteogenic progenitors and bone marrow stem cells that differentiate into osteoblasts. Subsequently, bone is formed under the hypertrophic layer (16,18). Histological observations of the present study show that the proliferative layer is thinner in diabetic rats than that of normal rats during experimental mandibular advancement. These result suggest that diabetes decreases chondrogenesis induced by mandibular advancement. In addition, transition from chondrogenesis to osteogenesis was also inhibited and subsequently decreased bone formation under the hypertrophic layer in the DG, as the hypertrophic layer was thicker at days 21 and 28 compared with that of the NG.

To further validate our hypothesis, the expression levels of MMP-8, MMP-9 and TIMP-1 were tested. Mandibular condylar cartilage is mainly consisted of chondrocytes and extracellular matrix (ECM), which is composed of proteoglycan aggregates and collagen fibre that contains collagen types I and II. The remodeling of condylar cartilage is characterized by the balance between the synthesis and degradation of various components of the ECM through the actions of MMPs. These actions may be inhibited by TIMP (19). MMP-8 (collagenases) and MMP-9 (gelatinases) are members of the MMP family, which cleave the major structural components of the ECM of condylar cartilage and may be inhibited by TIMP-1 (20). MMP-8 is expressed in chondrocytes of condylar cartilage throughout the cartilaginous cell layers during the growth period and ageing process, suggesting that it participates in the remodeling of the ECM during the differentiation of chondrocytes and degradation of cartilaginous ECM, accompanying endochondral bone formation (21). The results of the present study support this data and identified that mandibular advancement increases MMP-8 expression levels and the ratio of MMP-8 to TIMP-1 in condylar cartilage, while diabetes downregulates this augmentation. These results indicate that mandibular advancement enhances the remodeling of ECM and facilitates the proliferation of condylar chondrocytes and subsequent bone formation. Diabetes significantly impairs the remodeling of ECM and the proliferation of condylar chondrocytes caused by mandibular advancement.

Cartilage is usually resistant to vascular invasion (22). At the time of ossification, a specific process occurs to allow ingrowth of vessels into the matrix. MMP-9 is an important candidate for regulating these functions and is crucial for regulating hypertrophic cartilage angiogenesis by degrading the matrix and thus allowing penetration of endothelial cells and the release of specific factors that accelerate angiogenesis, for example vascular endothelial growth factor and fibroblast growth factor (23–25). In the present study, the mechanisms by which mandibular advancement increases the expression levels of MMP-9 in the proliferation and chondrogenic layers in the DG and NG, remain unknown, particularly as MMP-9 plays a crucial role in ossification. To understand this process, further analysis must be performed, taking into account whether MMP-9 participates in proliferation of condylar chondrocytes. Positive immunohistochemical staining for MMP-9 was observed in the hypertrophic layer at days 21 and 28 in the NG suggesting ossification occurred due to angiogenesis induced by MMP-9. Diabetes downregulated MMP-9 expression, the ratio of MMP-9 to TIMP-1 and the total layer thickness of the cartilage, which implies that diabetes may alter the condylar response to mandibular forward positioning. In addition, diabetes downregulated MMP-9 expression in the hypertrophic layer, indicating that diabetes may have impaired condylar ossification, which supports the histological findings that the hypertrophic layer was thicker than that of the NG at days 21 and 28.

According to our results, proliferation and ossification of condylar chondrocytes and subsequent bone formation in diabetic animals in response to mandibular advancement, was lower than that observed in healthy animals subjected to the same stimulus. Insulin deficiency-altering condylar growth modifications, which were induced by mandibular advancement, may account for these observations, since insulin exerts a direct effect on the chondrocytes and articular cartilage. Previous studies have shown that extracellular glucose concentrations may directly affect specific chondrocyte functions. For example, high glucose concentrations have been found to decrease proteoglycan synthesis and dehydroascorbate uptake, which is essential for collagen synthesis (26,27). High glucose levels also increased the expression of MMP-1 and MMP-13 in osteoarthritic human chondrocytes (28). Therefore, we hypothesize that hyperglycemia may also be associated with the alteration of condylar growth modification induced by mandibular advancement in diabetic animals. This hypothesis is consistent with the observations of the current study in which recovery of the condylar growth modification was observed in the TG, and subsequent blood glucose levels were well controlled.

In conclusion, the results of the present study suggest that young patients with type 1 diabetes mellitus with class II malocclusion who are not strictly monitored should not receive treatment with functional appliances until after hyperglycemia is controlled with insulin treatment.
